# Changes in Translational Control after Pro-Apoptotic Stress

**DOI:** 10.3390/ijms14010177

**Published:** 2012-12-21

**Authors:** Charline Lasfargues, Yvan Martineau, Corinne Bousquet, Stéphane Pyronnet

**Affiliations:** 1 INSERM UMR-1037 Université de Toulouse, Centre de Recherche en Cancérologie de Toulouse (CRCT) and Laboratoire d’Excellence Toulouse Cancer, TOUCAN, Toulouse 31432, France; E-Mails: charline.lasfargues@inserm.fr (C.L.); yvan.martineau@inserm.fr (Y.M.); corinne.bousquet@inserm.fr (C.B.); 2 Centre Hospitalier de Toulouse, Pôle Digestif, Toulouse 31432, France

**Keywords:** translation initiation, eIF2, eIF4F, 4E-BPs, apoptosis, uORF, IRES

## Abstract

In stressed cells, a general decrease in the rate of protein synthesis occurs due to modifications in the activity of translation initiation factors. Compelling data now indicate that these changes also permit a selective post-transcriptional expression of proteins necessary for either cell survival or completion of apoptosis when cells are exposed to severe or prolonged stress. In this review, we summarize the modifications that inhibit the activity of the main canonical translation initiation factors, and the data explaining how certain mRNAs encoding proteins involved in either cell survival or apoptosis can be selectively translated.

## 1. Introduction

Stress-induced apoptosis is a physiological mechanism of defense permitting the self-elimination of cells which cannot resolve damage caused by stress. The completion of apoptosis necessitates the realization of a specific gene expression program in which the control of protein synthesis plays an important role. When cells are exposed to an apoptotic stress, there is a substantial reduction in the global rate of protein synthesis, whereas in contrast, the synthesis of a subset of proteins is activated. Considerable progress has been made in understanding the molecular mechanisms responsible for such dual effects. The reduction in global translation rates has been attributed to changes in the activities of general translation initiation factors, while the selective induction of protein synthesis has been mainly assigned to the existence of specific regulatory sequences in certain messenger RNAs. These dual effects are viewed as adaptation processes permitting an appropriate response to stress intensity. Under mild and/or transient stresses, the inhibition of the global protein synthesis rate is indeed combined with the selective induction of protective proteins (such as ATF4 or CHOP under mild endoplasmic reticulum stress, see below) which resolve damage caused by stress. Upon severe and/or prolonged stress, pro-apoptotic proteins can be instead selectively induced (such as APAF-1, see below) to eliminate cells in which damage could be otherwise deleterious for tissue integrity.

To translate the genetic code into proteins, ribosomal (rRNA), transfer (tRNA) and messenger (mRNA) RNA species must be encountered. This process is part of the translation initiation step and is controlled in eukaryotes by a set of proteins called eukaryotic (translation) initiation factors (eIFs) [1]. One priming event is the formation of the 43*S* pre-initiation particle, a complex containing the 40*S* rRNA small ribosomal subunit and the tRNA. The binding of the tRNA to the rRNA is dependent on the activity of proteins which belong to group 2 of translation initiation factors (mainly the protein complexes eIF2 and eIF2B). Once the 43*S* pre-initiation complex is assembled, it must then bind to the mRNA, thus forming the 48*S* pre-initiation complex. This step is regulated by other components which belong to groups 3 and 4 of translation initiation factors (mainly the protein complexes eIF3 and eIF4F). During apoptosis and in stressed cells, the eIF2, eIF2B, eIF3 and eIF4F complexes are targeted by biological regulation, including changes in their phosphorylation status and their cleavage by caspases.

## 2. Inhibition of Global Protein Synthesis Due to Inactivation of Translation Initiation Factors

### 2.1. eIF2 and eIF2B

**Figure 1:**
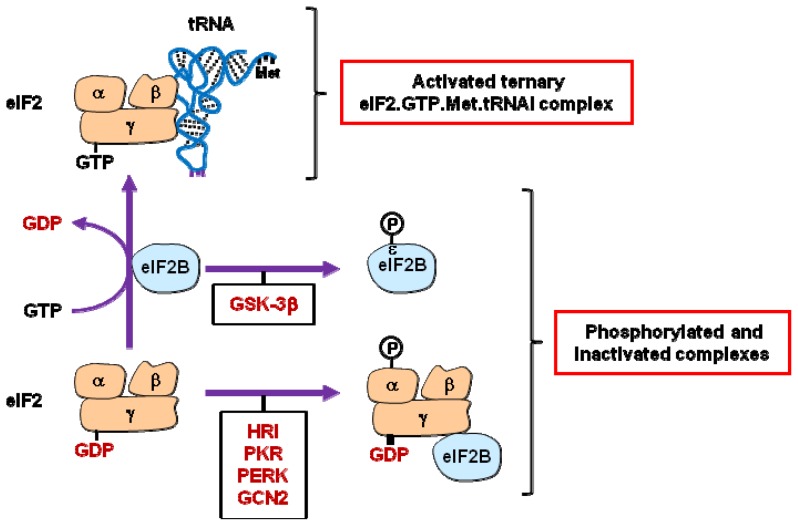
Regulation of eIF2 activity. The guanine nucleotide-exchange activity of eIF2B permits the association of the eIF2γ subunit of eIF2 with GTP. This is a prerequisite for the formation of the ternary eIF2.GTP.Met.tRNAi complex. eIF2 and eIF2B activities are inhibited by phosphorylation of their eIF2α (by HRI, PKR, PERK or GCN2) and eIF2Bɛ (by GSK3β) subunits. eIF2B activity is also indirectly inhibited (sequestered by eIF2γ) as a consequence of eIF2α phosphorylation. See text for details.

eIF2 is a heterotrimeric complex composed of three proteins: eIF2α, eIF2β and eIF2γ [2]. The tRNA can only interact with eIF2 once its eIF2γ subunit is bound to a GTP molecule. This is controlled by eIF2B, a translation initiation factor that possesses a guanine-nucleotide exchange activity (GEF or guanine nucleotide-exchange factor). However, upon phosphorylation of the eIF2α subunit, the GEF factor, eIF2B, harbors a higher affinity for GDP and is sequestered by the eIF2γ subunit. Since the interaction between the tRNA and eIF2 is a prerequisite for tRNA-rRNA assembly, the inhibition of tRNA interaction with eIF2 due to phosphorylation of the eIF2α subunit blocks protein synthesis (Figure 1). Four different protein kinases can phosphorylate eIF2α in mammals: HRI (heme-regulated inhibitor kinase), PKR (protein kinase RNA-activated), PERK (RNA-dependent protein kinase-like endoplasmic reticulum kinase) and GCN2 (general control nonrepressed 2). Three of them (PKR, PERK and GCN2) have been shown to be activated by various cellular stresses which may ultimately provoke apoptosis.

PKR is activated by ds (double-stranded) RNA [3]. This method of activation appears to be a mechanism of defense against viral infection. Upon viral infection, the generated dsRNA activates PKR which in turn phosphorylates eIF2α, blocking translation and limiting viral replication. dsRNA-dependent activation of PKR may also induce apoptosis of infected cells, another way to prevent viral spreading [4]. Since the initial discovery that PKR can trigger apoptosis [5], different molecular mechanisms have been described. PKR indeed elicits other routes which can induce apoptosis independently of eIF2α. For instance, in U937 cells, it seems that PKR-dependent activation of p53 is necessary for TNFα-induced apoptosis [6].

PERK is a protein that resides in the endoplasmic reticulum (ER) membrane. The cytoplasmic tail of PERK possesses an eIF2α kinase domain which is activated upon ER stress [7]. For instance, the activation of PERK and consequent phosphorylation of eIF2α occurs when the rate of synthesis of proteins destined for secretion exceeds the folding capacity of the ER [8]. This avoids the accumulation of misfolded proteins which could otherwise have deleterious effects for the cell. More generally, every stress that targets the ER (such as protein misfolding, oxidative stress or altered calcium pools) activates PERK, which in turn blocks protein synthesis via eIF2α phosphorylation [9].

GCN2 is the unique eIF2α kinase present in all eukaryotes from yeast to mammals [10]. Although it was initially characterized as a sensor of amino acid availability in yeast [11], GCN2 can also be activated either upon deprivation of other nutrients such as glucose [12] and oxygen [13], or under other stresses such as exposure to UV light [14].

In addition to its phosphorylation, eIF2α has been shown to be the target of another post-transcriptional modification. Once the apoptotic program has been engaged, eIF2α itself becomes a caspase substrate. Caspase 3-mediated truncation generates a shorter eIF2α protein which can still interact with eIF2B yet is no longer capable of stimulating overall translation [15,16], further supporting the importance of eIF2α inactivation in the completion of apoptosis.

eIF2B is a heteropentamer whose ɛ catalytic subunit promotes GDP/GTP exchange on eIF2. Consistently, eIF2B activity is negatively regulated when its eIF2Bɛ subunit is phosphorylated by the protein kinase GSK3β (glycogen synthase kinase 3β) [17] (Figure 1). Inhibition of eIF2B activity due to eIF2Bɛ phosphorylation by GSK3β has, however, been shown to be relieved upon activation of the PI3K-AKT pathway, thus leading to cell survival. Conversely, eIF2Bɛ phosphorylation by GSK3β appears to be involved in programmed cell death, triggered by amino acid or growth factor deprivation, which inhibits PI3K-AKT signaling [17,18]. More recently, the importance of eIF2B activity in the regulation of the cell survival/apoptosis balance has been further highlighted. CHOP (C/EBP-homologous protein, also known as GADD153)-dependent inhibition of protein synthesis and consequent apoptosis, due to phosphorylation of eIF2α can, in fact, be counteracted by PP2A (protein phosphatase 2A)- mediated dephosphorylation of eIF2Bɛ [19].

These data indicate that inhibition of protein synthesis and induction of apoptosis might be fully achieved solely when both eIF2 and eIF2B are inactivated, via concomitant phosphorylation of their α and ɛ subunits, respectively (Figure 1).

### 2.2. eIF3 and eIF4F

Changes in eIF2 and eIF2B activities are not the only pathways utilized by the cell to limit protein synthesis upon stress. The attachment of the eIF2.GTP-containing 43*S* particle to the mRNA is also a tightly regulated molecular process. This regulation is mainly dependent on the eIF3 and eIF4F translation initiation factors whose activities are targeted by various pro-apoptotic stresses.

eIF3 is a protein complex (composed of 13 polypeptides in mammalians, termed eIF3e-m) which links the mRNA-cap-binding factor eIF4F (via its eIF4G component, see below) to the tRNA-containing 43*S* ribosomal subunit. One of the first indicators of a stress-inducible inhibition of eIF3 activity has been obtained in virus infected cells. Indeed, the binding of eIF3 to either the 43*S* particle or the eIF4F complex has been shown to be disrupted following viral infection [20]. Alterations of these complexes were attributed to interactions of eIF3 subunits (eIF3e or eIF3c) with members of the virus-induced ISG56/IFIT1 (interferon-stimulated gene 56 kDa/interferon-induced protein with tetratricopeptide repeats 1) family of defense proteins [21]. The virus-induced and ISG56/IFIT1-dependent inactivation of eIF3 and consequent inhibition of mRNA translation is similar to that described for the virus-induced and PKR-dependent inhibition of eIF2 activity, and therefore can be viewed primarily as a defense against viral replication. The anti-apoptotic role of eIF3 was later documented in non-infected cells. Five subunits of eIF3 (a, b, c, h and i) have been shown to be overexpressed in various tumors and their overexpression in cellular models actually promotes transformation, in part through induction of cell viability [22]. Conversely, inhibition of protein synthesis due to eIF3 inactivation may contribute to the execution of apoptosis, since at least one subunit of eIF3 (eIF3j) is a caspase substrate [23]. Other signals which inactivate eIF3 and promote apoptosis have been reported. The phosphorylation of the eIF3f subunit by the caspase-activated form of CDK11 (cyclin-dependent kinase 11) is also noteworthy [24]. Another example of eIF3 promoting apoptosis is the re-localization of the eIF3k subunit to keratin intermediate filaments, where the factor frees active caspase 3 from retention by keratin 8, when epithelial cells are exposed to apoptotic stimuli [25].

**Figure 2:**
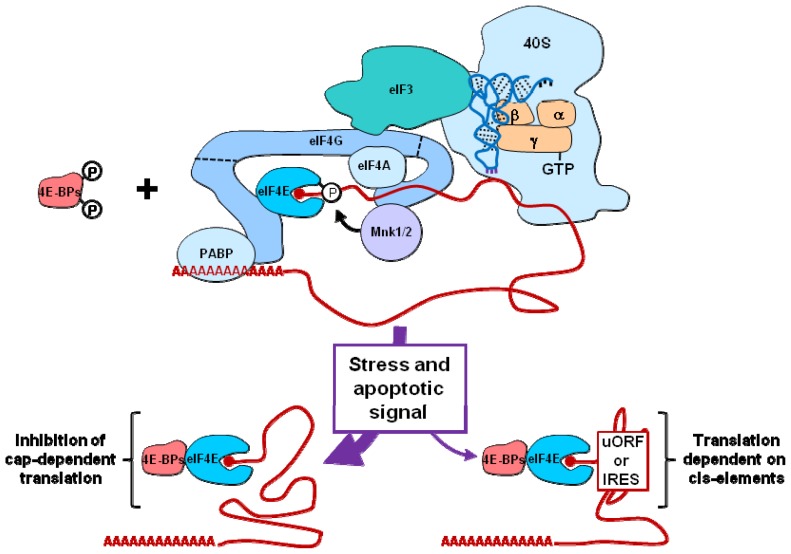
Translational functions of the eIF3 and eIF4F complexes and inhibition by the 4E-BPs. The eIF4F complex is composed of eIF4E, eIF4G and eIF4A. The interaction between eIF4F (bound to the cap structure via its eIF4E subunit) and eIF3 (bound to the 40*S* ribosomal particle) complexes permits ribosome docking at the 5′ extremity of capped mRNAs. The melting of mRNA structures by the RNA helicase eIF4A facilitates ribosome binding to mRNA, and the interaction between eIF4G and PABP results in mRNA circularization, a feature that is believed to enhance mRNA translation. eIF4G recruits the eIF4E kinases MNK1 and MNK2. In stressed cells, the dephosphorylation of 4E-BPs disrupts the eIF4E-eIF4G interaction, inhibiting general cap-dependent translation (left arrow). However the translation of selected mRNAs is still possible due to the existence of either uORFs or IRESes (right arrow). See text for details.

eIF4F is a heterotrimeric translation initiation factor composed of eIF4E, eIF4A and eIF4G (Figure 2). eIF4E recognizes and binds the mRNA 5′ cap structure. eIF4A possesses an RNA-helicase activity that facilitates the interaction between the 43*S* complex and the mRNA by melting mRNA 5′ end secondary structures. eIF4G is a scaffolding protein which simultaneously binds eIF4E, eIF4A and eIF3, thus providing a physical link between the 43*S* particle and the mRNA [26]. eIF4G also interacts with the two protein kinases that phosphorylate eIF4E, the MAPK-integrating protein kinases 1 and 2 or MNK1 and MNK2 [27,28]. These two protein kinases lie downstream of the Ras/MEK pathway. Activity of the eIF4F complex is drastically inhibited in the process of apoptosis by signals that target phosphorylation and/or integrity of its three subunits (eIF4E, eIF4A and eIF4G).

One priming event leading to eIF4F inhibition which is triggered by apoptotic signals is the association of eIF4E with the hypophosphorylated forms of one of its inhibitory proteins, called 4E-BP1 and 4E-BP2 (eIF4E-binding proteins 1 and 2) [29]. Indeed, the hypophosphorylated forms of 4E-BP1 and 4E-BP2 compete with eIF4G for a common binding site on eIF4E (Figure 2). In surviving/proliferating cells, 4E-BP1 and 4E-BP2 are normally hyperphosphorylated by the PI3K/AKT/mTOR pathway. However, upon various stresses (such as amino-acid deprivation, hypoxia, or DNA-damage) and during apoptosis, 4E-BP1 and 4E-BP2 are hypophosphorylated due to inhibition of the PI3K/AKT/mTOR pathway. 4E-BP1 hypophosphorylation results not only from downregulation of the mTOR axis, but also from a cleavage by caspases that removes the RAIP-containing *N*-terminus of 4E-BP1. The RAIP motif (named after its sequence) is normally required for optimal phosphorylation of 4E-BP1 (and also 4E-BP2). The resulting truncated form of 4E-BP1 acts as a dominant inhibitor of cap-dependent translation [30]. In addition, as MNK1 and MNK2 use a docking site in eIF4G to facilitate eIF4E phosphorylation, disruption of the eIF4E-eIF4G complex by stress-mediated hypophosphorylation of 4E-BP1 and 4E-BP2 is expected to negatively impinge upon eIF4E phosphorylation (Figure 2).

Two functional homologs of eIF4A (termed eIF4AI and eIF4AII) have been characterized [31]. Their activity is specifically controlled by PDCD4, the programmed cell death protein 4. PDCD4 is induced in most cells that undergo an apoptotic program, whereas the protein is downregulated in transformed cells. Consistently, the forced expression of PDCD4 exerts an anti-tumoral effect [32]. Such an anti-tumoral effect is mediated in part through inhibition of protein synthesis since PDCD4 interacts with and sequesters eIF4AI or eIF4AII away from cap-dependent translation initiation complexes [33]. Another mechanism that can account for inhibition of eIF4AI or eIF4AII activity in apoptotic cells is their sequestration by fragments of eIF4G generated by caspase cleavage (see below).

Two functional homologs of eIF4G (termed eIF4GI and eIF4GII) have also been described [34]. The two proteins are, however, subjected to different changes (described below) upon induction of programmed cell death. High rates of protein synthesis are correlated with phosphorylation of eIF4GI by the PI3K/AKT/mTOR pathway. Consistently, stress and apoptotic signals that inhibit this pathway are also expected to inhibit eIF4GI phosphorylation. Interestingly, cells have evolved another mechanism independent of 4E-BP1 and 4E-BP2 to disrupt the eIF4E-eIF4G interaction. Under stress, eIF4G actually associates with and is phosphorylated by PAK2 (p21-activated protein kinase 2). The PAK2 binding site on eIF4G overlaps with that of eIF4E, therefore PAK2 and eIF4E compete with each other for interaction with eIF4G. eIF4G phosphorylation by PAK2 further inhibits translation initiation [35]. The most significant change that affects eIF4GI in the process of apoptosis is certainly its cleavage by caspases. The cleavage of eIF4GI at two main sites by caspase 3 produces three fragments which have been named N- M- and C-FAG (N, M and C for *N*-terminus, middle and *C*-terminus; FAG for fragment of apoptotic cleavage of eIF4GI) (Figure 2). Interestingly, the resulting fragments retain their capability to interact with eIF4GI partners and therefore can form sub-complexes which could be intuitively classified as either translation inhibitory or activating sub-complexes. N-FAG contains the poly(A)-binding protein (PABP, see below) binding domain while C-FAG possesses eIF4A and MNK1 or MNK2 binding domains. Through sequestration of PABP, eIF4A and MNK kinases, it could be anticipated that these sub-complexes act as inhibitors of translation initiation. M-FAG encompasses the binding domains for eIF4E, eIF4A and eIF3. These features have been shown to constitute a core eIF4GI, capable of both recruiting the 43*S* particle (via eIF3) to the mRNA 5′ end (via eIF4E) and facilitating the ribosome binding to the mRNA (via eIF4A). However, experimental evidence indicates that M-FAG does not support translation initiation in apoptotic cells [36]. Therefore, caspase-dependent cleavage of eIF4GI appears to mainly participate in the general inhibition of protein synthesis observed in apoptotic cells.

eIF4GII is also a substrate of caspase 3. At least five different caspase cleavage sites have been mapped in the eIF4GII protein sequence [37]. In contrast to eIF4GI, this extensive cleavage does not produce a “core” eIF4GII and instead generates smaller fragments which either render eIF4GII inactive in translation initiation or can even act as dominant inhibitors by sequestering eIF4Gs’ partners. Interestingly, the cleavages of eIF4GI and eIF4GII by caspase 3 have been shown to occur with similar kinetics and correlate with the profound shutdown of protein synthesis observed in apoptotic cells [37].

As mentioned above, the *N*-terminal thirds of eIF4GI and eIF4GII interact with PABP. The resulting mRNA circularization (Figure 2) has been shown to stimulate translation initiation [38]. Therefore, PABP sequestration by caspase-generated *N*-terminal fragments of eIF4GI and eIF4GII may participate in the translation inhibition observed in apoptotic cells. Degradation of PABP has also been shown in apoptotic cells through a mechanism partially dependent on caspase inhibitors [39].

A distant homolog of eIF4GI and eIF4GII, called p97 or DAP5 (death-associated protein 5), also exists (hereafter named p97) [40]. In contrast to eIF4GI and eIF4GII, this factor does not possess the binding sites for PABP and eIF4E. However p97 does contain the binding sites for eIF4A and eIF3, and several studies have reported that, although not important for general translation, p97 function is required for regulating the expression of specific genes implicated in cell proliferation [41] and differentiation [42]. In addition, p97 is cleaved by caspases during apoptosis, yielding an *N*-terminal fragment that still binds eIF4A and eIF3 [43], and controls the expression of anti- or pro-apoptotic genes (see below).

Thus, many of the translation initiation factors are inactivated before and during apoptosis. The inhibition of protein synthesis observed in apoptotic cells is therefore a multifactorial process in which the individual contribution of every translation initiation factor appears difficult to dissect. However, there has been an improved exploration into how certain mRNAs are selectively translated despite the inactivation of global protein synthesis.

## 3. Selective Translation of mRNAs Encoding Pro-Survival or Pro-Apoptotic Proteins

The inhibition of global translation rates, as a result of various stresses, appears to primarily exert a cytoprotective effect. Indeed, as protein synthesis is a very energy-consuming process, it can be assumed that stress-induced inactivation of eIF2, eIF2B, eIF3 and eIF4F complexes is an adaptation mechanism that has evolved both to save energy and facilitate an appropriate stress-response. The destiny of stressed cells (survival or death) is dictated by the activity of pro-survival or pro-apoptotic proteins whose synthesis may paradoxically depend on translational events. The synthesis of these proteins is possible due to their mRNAs containing specific *cis*-acting elements that permit ribosome recruitment, despite the general inhibition of protein synthesis.

### 3.1. uORF-Dependent Translation

One of the mechanisms of apoptosis most widely referred to is the induction of cell death by ER stress triggered by eIF2α phosphorylation. ER-stress-induced apoptosis is indeed dependent on ATF4 (activating transcription factor-4), whose mRNA translation is specifically enhanced following eIF2α phosphorylation. In mammalian cells not subjected to stress, ATF4 mRNA translation is kept silenced due to the presence of two short uORFs (upstream open reading frames) [7] located in the 5′ untranslated region (UTR). In the absence of eIF2α phosphorylation, ribosomes which have translated the first uORF can efficiently re-initiate at the second uORF, thus precluding translation initiation at the downstream ATF4 ATG codon. However, upon ER stress and consequent eIF2α phosphorylation, initiation at the second uORF is impaired due to the decrease in activated ternary complex. Ribosomes which have bypassed the second uORF then scan to the downstream ATF4 initiator ATG. Such scanning offers a larger window in which the ternary complex can re-activate so that translation of the ATF4 ORF can occur [44,45]. Among the transcriptional targets of ATF4, the transcription factor CHOP plays a prominent role. Interestingly, CHOP mRNA can also be translated under ER stress as eIF2α phosphorylation permits ribosome bypass of an inhibitory uORF [46]. ATF4 and CHOP, usually through interaction with other transcriptional regulators, then participate in ER-stress-corrective actions through the modulation of a number of genes. When cells are recovering from stress, eIF2α phosphorylation returns to basal low levels, in part through a GADD34- (growth arrest and DNA damage-inducible protein 4) and the protein-phosphatase-1-mediated process. Interestingly, GADD34 expression is also dependent on a uORF whose translation inhibitory role is prevented by eIF2α-phosphorylation [47]. However, severe or prolonged stress can ultimately induce apoptosis through a process that can also be dependent on eIF2α phosphorylation and consequent induction of both ATF4 and CHOP [48]. eIF2α-, ATF4- and CHOP-triggered apoptosis has been linked to changes in the expression of pro- or anti-apoptotic molecules, such as suppression of the pro-survival protein Bcl-2 (B-cell lymphoma 2) [49] and induction of pro-apoptotic DR5 (death receptor-5 also termed TRAIL-R2) [50] or TRB3 (Tribbles-related protein 3) [51].

The induction of translation upon eIF2α phosphorylation does not apply to all uORF-containing mRNAs. For instance, the 5′ UTR of HIAP2 (human inhibitor of apoptosis protein) mRNA contains a uORF which inhibits translation in proliferating cells [52] yet is not involved in increased translation upon stress. Instead, the translation of HIAP2 mRNA in stressed cells is induced by the presence of another *cis*-acting element [53].

### 3.2. IRES-Dependent Translation

As mentioned above, inactivation of eIF3 and eIF4F complexes and consequent inhibition of ternary complex fixation to the mRNA 5′ cap largely participate in the downregulation of global translation rates when cells are exposed to stress. However, the translation of certain mRNAs can be induced if they contain a specific sequence called an IRES (internal ribosome entry site), which permits the recruitment of the ribosome independently of the cap structure. These IRES-containing mRNAs encode many proteins that protect against apoptosis, although in contrast a few of them can facilitate the execution of apoptosis.

The most documented examples of cell-stress-induced IRESes are those of the mRNAs encoding proteins which belong to the IAP (inhibitor of apoptosis protein) and Bcl-2 families of anti-apoptotic proteins (Table 1) including HIAP2 [53], XIAP [54], c-IAP1 [55], Bcl-2 [56] and Bcl-xL [57]. IRES-mediated translation of the mRNAs coding for HIAP, XIAP and Bcl-2 is dependent on the eIF4G homolog, p97. The p97 protein lacks an eIF4E-binding site and is involved in the recruitment of translation initiation complexes to IRESes independent of both eIF4E and, consequently, the inhibition of cap-dependent translation due to 4E-BPs hypophosphorylation. Interestingly, p97 mRNA itself possesses an IRES which permits p97 protein expression under stress conditions [43]. In addition, when the apoptotic program has been engaged, the cleavage of p97 by caspases generates a shorter p86 protein fragment that further activates its function in IRES-mediated translation. One mRNA whose IRES-mediated translation is enhanced by the caspase-generated p86 fragment is that of Apaf-1 (apoptotic peptidase activating factor 1), a component of the apoptosome [36,58]. Thus, through its different mRNA targets, p97 appears to play a dual role. This factor plays primarily a protective function via the translation of mRNAs encoding pro-survival proteins, while under severe or prolonged stress it may exert a pro-death function via the induction of pro-apoptotic proteins.

Other mRNAs have been shown to possess IRESes which function under stress or apoptosis. They encode proteins belonging to various biological functions. Among them, the transcriptions factors cMyc [59] and Sp1 [60], the cyclin-dependent kinase 1 CDK1 [61], the chaperone Bip [62] and the amino-acid transporter Cat-1 [63] exert pro-survival effects, while the protein kinase C delta (PKCδ) [64] exerts instead a pro-apoptotic activity (Table 2).

## 4. Conclusions

As mentioned above, most of the mRNAs identified to date as being translated under stress, despite a general inhibition of protein synthesis, contain specific sequences (uORF or IRES) and encode proteins involved in cell survival. The list of uORF-containing mRNAs appears relatively limited. It is probable, however, that the fast developing techniques of high throughput RNA sequencing will help to identify other mRNAs possessing such regulatory sequences. Similarly, IRES-mediated translation in stressed cells is probably underestimated. In this regard, an elegant study aimed at identifying the translational profile of MCF7 cells treated with the apoptosis-inducing ligand TRAIL, indicated that approximately 3% of mRNAs remain associated with the polysomes in apoptotic cells [65]. Although a certain number of these mRNAs were already known to contain an IRES (*i.e.*, c-Myc), novel functional IRESes were identified, particularly in mRNAs encoding proteins required for chromatin modification/remodeling and Notch signaling. These findings suggest that the list of stress- or apoptosis-regulated uORF- or IRES-containing mRNAs should be extended in the near future. This will certainly help to clarify how cells are capable of modifying gene expression at the translational level to better cope with stress.

Another feature that cells have evolved to selectively regulate gene expression at the mRNA stability/translational level is the use of micro-RNAs (miR). As shown above, the existence of uORFs and IRESes in mRNAs often permits a selective induction of protein synthesis. In the case of micro-RNAs, the expression of target genes is instead selectively inhibited, as was shown for the anti-apoptotic Bcl family of proteins, whose Bcl-2, Mcl-1, and Bcl-xL members are targeted by miR 15–16 [66], miR 29b [67] and Let-7/mir 98 [68], respectively. However, how the expression of these micro-RNAs is up- or downregulated by apoptotic stress remains to be fully characterized.

## Figures and Tables

**Table 1:** Modification of translation initiation factors both prior to and during apoptosis and their involvement in inhibition of general cap-dependent translation (middle column) and selective translation dependent on *cis*-elements (right column).

Translation initiation factor	Modifications of initiation factors involved in inhibition of cap-dependent translation	Involvement of modified initiation factors in selective translation dependent on cis-elements
**eIF2**	.Phosphorylation of eIF2α [5,8–10]	.Relief of uORF-mediated translation initiation [44–47]
	.Caspase cleavage of eIF2α [15,16]	

**eIF2B**	.Phosphorylation of eIF2Bɛ [17–19]	

**eIF3**	.Caspase cleavage of eIF3j [23]	
	.Phosphorylation of eIF3f by CDK11 [24]	
	.Interaction of eIF3c or eIF3e with ISG56/IFIT1 [21]	
	.Sequestration by eIF4G caspase cleavage fragments [36]	

**4E-BP1/2**	.Caspase cleavage of 4E-BP1 [30]	

**eIF4F**		
eIF4E	.Sequestration by hypophosphorylated and cleaved 4E-BP1/2 [29,30]	
	.Sequestration by eIF4GI/II-caspase-cleavage fragments [36]	
eIF4AI/II	.Interaction with PDCD4 [33]	
	.Sequestration by eIF4GI/II-caspase-cleavage fragments [36]	
eIF4GI/II	.Phosphorylation of eIF4GI by PAK2 [35]	
	.Caspase cleavage of eIF4GI and eIF4GII [36]	

**PABP**	.Caspase cleavage [39]	
	.Sequestration by eIF4GI/II-caspase-cleavage fragments [36]	

**P97/DAP5**		.Induction of IRES-mediated translation [53–55]

**Table 2:** List of uORF- or IRES-containing mRNAs that are translated in stressed or apoptotic cells and which encode either cell survival (green) or cell death (red) proteins.

	uORF		IRES		
		
Function	Survival	Death	Survival	Death	References
Transcription	ATF4				[45]
	CHOP				[46]
			c-Myc		[59]
			Sp1		[60]

IAP family (survival)			HIAP		[53]
			XIAP		[54]
			c-IAP		[55]

Bcl family (survival)			Bcl-2		[56]
			Bcl-x(L)		[57]

Apotosome				APAF-1	[58]

Cell cycle			CDK1		[61]

Chaperone			Bip		[62]

Translation			P97/DAP5	P97/DAP5	[43]

Signaling	GADD34				[47]
				PKCδ	[64]

Amino-acid transport	Cat-1		Cat-1		[63]
